# The Effects of Menorrhagia on Women's Quality of Life: A Case-Control Study

**DOI:** 10.1155/2013/918179

**Published:** 2013-07-08

**Authors:** Sule Gokyildiz, Ergul Aslan, Nezihe Kizilkaya Beji, Meltem Mecdi

**Affiliations:** ^1^Midwifery Department, Cukurova University Adana Health High School, Balcali Kampusu, Saricam, 01330 Adana, Turkey; ^2^Istanbul University Florence Nightingale Faculty of Nursing, Obstetrics and Gynaecology Nursing, Sisli, 34387 Istanbul, Turkey; ^3^Obstetrics and Gynaecology Clinic, Istanbul Faculty of Medicine, Istanbul University, Sehremini, 34104 Istanbul, Turkey

## Abstract

*Objective*. The purpose of this study is to identify menstruation characteristics of the women and the effects of menorrhagia on women's quality of life. *Methods*. The study was designed as a descriptive, case-control one. *Results*. Of the women in the case group, 10.9% stated that their menstrual bleeding was severe and very severe before complaints while 73.2% described bleeding as severe or very severe after complaints. Among those who complained about menorrhagia, 46.7% pointed that they used hygienic products that are more protective than regular sanitary pads. Women also stated that their clothes, bed linens, and furniture got dirty parallel to the severity of the bleeding. In all subscales of SF-36 scale, quality of life of the women in the menorrhagia group was significantly lower than the ones in the control group (*P* < 0.05). *Conclusion*. Menorrhagia has negative effects on women's quality of life. Therefore, quality of life of the women consulting the clinics with menorrhagia complaint should be investigated and effective approaches should be designed.

## 1. Introduction

Menorrhagia, one of the most frequently encountered symptoms in gynaecology, is defined as menstruation periods at regular cycle but with excessive flow which may last more than 7 days. Menorrhagia can cause menstrual bleeding of more than 80 mL in each cycle [1]. 

Menorrhagia is a major cause of gynaecological diseases that affect 1–5 women living in Europe and North America in a period of their reproductive age; 9–14% of women in their reproductive age lose 80 mL blood in each cycle. This proportion shows similar frequency in developing countries as well. It was indicated that 12% of the adolescents in Nigeria complained about menorrhagia with blood loss over 80 mL. As to our country, 16% of the women aged between 15 and 44 were diagnosed with menorrhagia, and 25% of the women complained about long-frequent periods of bleeding or staining. In its multiple country study, World Health Organization (WHO) identified the prevalence of three-month severe bleeding as 8–27% [2]. 

Quality of life is the perception of individuals' situations in life in relation to their aims, expectations, and standards within the framework of their cultural and value systems [3]. Despite rarely being life-threatening, menorrhagia has significant effects on personal, social, family, and work life of women and thereby reduces their quality of life [4]. Women describe the loss or reduction of daily activities as more important than the actual volume of bleeding [5]. Menorrhagia is largely responsible for iron deficiency and iron deficiency anaemia both of which have negative effects on women health, women's consulting gynaecology departments, being hospitalized, and having operation. Several studies mention the negative effects of menorrhagia on women [4, 6–9].

Studies on heavy menstrual bleeding seem to focus traditionally on the measurement of blood loss which is not clinically so significant; they usually fail to evaluate patients' experience and self-evaluation [10, 11]. In their focus group study, Matteson and Clark [7] (2010) emphasize the importance of patients' self-evaluation regarding their experience, blood loss, and its effects on their lives in diagnosing as well as planning treatment for women with abnormal uterine bleeding. 

Studies on menorrhagia conducted in our country are about treatment [12, 13]. Our study is the first case and control group study in relation to menstruation characteristics and quality of life of women with menorrhagia.

## 2. Aim

In this study, we aim to identify menstruation characteristics of the women and the effects of menorrhagia on women's quality of life. 

## 3. Methods

### 3.1. Design

We designed the study as a descriptive, case-control study. 

### 3.2. Participants

The participants are 295 volunteer women who were not pregnant or had menopause at the time the study was conducted and who consulted to the Department of Gynaecology and Obstetrics at a University Hospital between January 2008 and January 2010. The patients who had menorrhagia complaint were included in the case group (*n* = 138) while the relatives of the participants who did not have any specific health problems composed the control group (*n* = 157). 

### 3.3. Instruments

We collected data via face-to-face interviews with a questionnaire form prepared by the authors in light of the related literature and SF-36 Quality of Life Scale [14]. The questionnaire form consisted of 30 questions regarding women's sociodemographical (age, education, occupation, and financial situation) features, obstetrics (pregnancy and number of birth) and menstruation characteristics, and gynaecological and medical problems (see the Appendix). The SF-36 questionnaire consists of 36 items covering eight distinct health status concepts and one item measuring self-reported health transition: physical functioning, physical role functioning, pain, general health, vitality, social role functioning, emotional role functioning, and mental health. The quality of life increases as the score of each aspect in the scale increases [14]. The scale was adapted to Turkish society by enhancing its reliability and validity in Pınar's [15] (1995) study with diabetics.

We administered the questionnaires while the women were waiting for their clinic visit. The women in the case group compared the questions about menstruation characteristics before and after menorrhagia.

### 3.4. Ethical Considerations

We obtained the written ethical approval from the ethical review board of the university where we conducted the study. The participants were informed about our aims in the study and their verbal consent was obtained prior to the administration of the questionnaire. 

### 3.5. Data Analysis

We analyzed the data obtained from the study using SPSS (Statistical Programme for Social Science) 11,5 for Windows and evaluated them through frequency, mean, standard deviation, chi-square, Wilcoxon Rank, and Mann-Whitney *U* test [16]. 

## 4. Results

We found no significant differences between women in control and case groups in terms of age, education, occupation, financial situation, pregnancy and number of birth, general health problems, and using drugs. We found the average age of the participants in the case group as 35.86 ± 8.67 while that in the control group as 32.18 ± 8.49. The majority of the women in both groups received education for 5–8 years, and they were housewives. In addition, 21% of the women in the case group did not have any children, 16.7% had one child, 32.6% had two children, 13.8% had three children, and 15.9% had four and more children. As to those in the control group, 23.9% had no children, 17.7% had one child, 31.5% had two children, 14.1% had three children, and 12.8% had four and more children. 

We found the duration of menorrhagia complaints as follows: 18.8% (*n* = 26) of the women in the case group for three months or less, 20.3% (*n* = 28) for 4–7 months, 8% (*n* = 11) for 8–11 months, 14.5% (*n* = 20) for 1-2 years, 14.5% (*n* = 20) for 2-3 years, and 23.9% (*n* = 33) had been suffering from menorrhagia for more than three years. Of these women, 34.8% (*n* = 48) had treatment, and a great majority was given medication (*n* = 43). We found the diagnosis for the women in the case group as myoma for 26.1%, genital tract infection for 8.7%, polyp for 5.8%, endometrial hyperplasia for 3.6%, and endometritis for 2.2%. 

Women in the case and control groups indicated that they did not know of any specific illness which causes the bleeding problem. Of the participants, 41.3% from the case group and 27.3% of those in the control group pointed that there was somebody in their families with menorrhagia complaint. We found that the participants' relationship with these women for the women in the case group was as follows: mother: 18.1%, sister: 15.2%, and aunts: 7.9%, as to control group; mother: 17.8%, sister: 7.6%, and aunts: 1.9%. Women in both groups pointed that there was not any other woman in their families with bleeding problem. 

We found average length of menstrual cycle before menorrhagia as 28.11 ± 4.86 days for the women in the case group and as 22.79 ± 7.27 days after menorrhagia. We identified average length of menstrual cycle for the women in the control group as 28.13 ± 5.80 days. We also found that there was a significant difference in the average menstrual cycle of the women in the case group before and after menorrhagia and between the case and control groups. 

**Figure 1:**
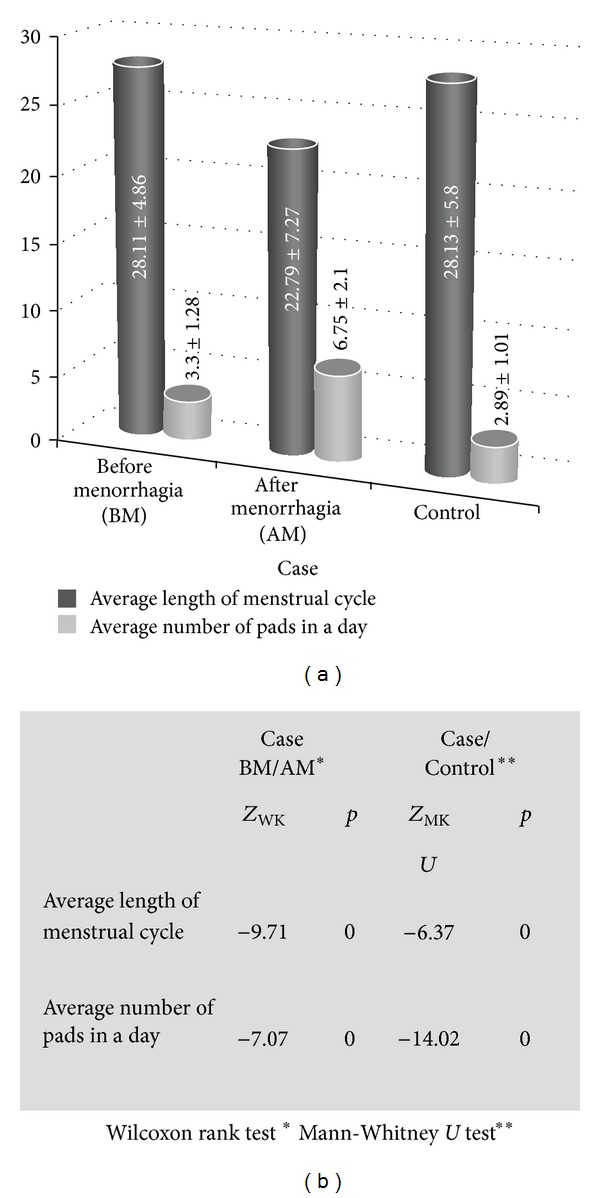
Average length of menstrual cycle and average number of pads in a day of the participants.

 Women in the case group reported to have used 3.30 ± 1.28 pads on the average before menorrhagia, and 6.75 ± 2.10 pads after menorrhagia while the women in the control group reported to use 2.89 ± 1.01 pads on the average. We found a significant difference between before menorrhagia and after menorrhagia for the case group and between case and control groups in terms of the average number of pads used during one cycle. We also found that women in the case group displayed a decrease in their cycle duration and an increase in the number of pads used after menorrhagia (Figure 1).

Table 1 displays findings regarding the participants' menstruation characteristics. Of the women in the case group, 89.1% (*n* = 123) stated that the menstrual bleeding was mild and moderate before complaints while 10.9% (*n* = 15) described the bleeding as severe and very severe. After complaints, the bleeding was described as mild and moderate by 26.8% (*n* = 37) and severe and very severe by 73.2% (*n* = 101). As to those in the control group, 91% (*n* = 142) described their menstruation bleeding as mild and moderate, and 9% (*n* = 14) as severe and very severe. Among those who complained about menorrhagia, 46.7% pointed that they used hygienic products that are more protective than regular sanitary pads. Women also stated that their clothes, bed linens, and furniture got dirty parallel to the severity of the bleeding. We found that there was an increase in the pain together with the increase in menorrhagia. The comparison of the participants in terms of their menstruation characteristics demonstrates that there are statistically significant differences between the case group and control group (*P* < 0.05). 

We evaluated the participants' quality of life and found that menorrhagia group members were affected more significantly in all subscales of the SF-36 scale (physical functioning, physical role functioning, pain, general health, vitality, social role functioning, emotional role functioning, and mental health) when compared to the women in the control group (Table 2). 

## 5. Discussion

Menorrhagia is considered to be one of the most significant causes of ill health in women. One in 20 women aged between 30 and 49 years consults her general practitioner each year with heavy menstrual loss [17]. More than half of the women in the case group (52.9%) reported to have had menorrhagia for more than a year. 

Studies show that although menorrhagia rarely threatens life, it has negative effects on women's personal, family, social, and work life and it decreases quality of life [4, 6, 7, 18–20]. Shankar et al. [9] (2008) conducted a review of studies evaluating quality of life in women suffering from menorrhagia. In their systematic review, they indicate that health related quality of life is adversely affected in women with menorrhagia in general and in those with inherited bleeding disorders [9]. Studies which aim to identify quality of life make use of instruments such as SF-36, SF-12, or Euro QOL-5D [9]. Studies that have used SF-36 in women with menorrhagia show that all subdimensions of the scale indicate low scores [21, 22]. Similar to the findings in the literature, we have found that menorrhagia affects women's quality of life in a negative way, and this effect reveals itself in all eight subdimensions of SF-36 Quality of Life Scale which includes functioning, pain, general health, vitality, social role functioning, emotional role functioning, and mental health.

A careful anamnesis is an important factor in evaluating patients with menorrhagia complaint as well as exploring the underlying reasons [23]. Menorrhagia can be associated with fibroids, endometriosis, adenomyosis, cervical or endometrial malignance, intrauterine devices, or pelvic infection. Sometimes it can be caused by factors in relation to hypothyroidism or bleeding illnesses [5, 24, 25]. We found no significant differences between the case and control groups in terms of the participants' women health and general health problems. Women in both case group and control group reported that they did not know of any specific disease that caused menorrhagia, but the ones in the case group were diagnosed with such diseases as myoma, genital tract infection, polyp, endometrial hyperplasia, and endometritis. 

Menorrhagia diagnosis and blood loss can be identified by making use of various methods such as women's own statements, menstruation duration, the number of sanitary pads used in each menstruation, weight of sanitary pads in each menstruation, laboratory analysis of the blood content of used sanitary products, and the Pictorial Blood Loss Assessment Chart [4, 8]. Although the definition of menorrhagia includes menstrual bleeding that lasts more than seven days, this definition is not valid by itself [4, 8]. The number of menstruation days is not important in diagnosing menorrhagia; we found that the women began to have menstruation in shorter intervals. 

It is self-evident that the number of sanitary pads used will be more during heavy menstruation periods than lighter ones. On the other hand, hygiene habits of women and their financial situation also have effects on the number of pads used [4, 8]. Through a comparison of the case group before and after menorrhagia as well as with the control group in terms of the number of pads used, we found that there was an increase in the number of pads used after menorrhagia.

In their ethnographic study, Kinnick and Leners [20] (1995) conducted in-depth interviews with 6 women three months after elective hysterectomy. The first result obtained from the data analysis was the term “miserable”; all the women described their preoperative problems as “… making them feel miserable.” Women described their menstrual bleeding using the term “gush” and further explained their states as “having to leave work and go home to change their clothes” or as “the adventure of setting the alarm clock at intervals throughout the night so as to avoid drenching the bed” [20]. We found that the preoperative complaints of the women had tremendous effects on women's quality of life and there were positive changes in their complaints after hysterectomy as women described the changes in their lives using the expressions “very good” and “great.” In their study with 767 university students aged between 18 and 39, Anastasakis et al. [26] found that 35% (*n* = 268) of the students had severe menstruation and 60% of them reported to have negative effects on their quality of life. 87.7% (*n* = 235) of the women participating in the study stated that their clothes got dirty during menstruation, and 55.2% (*n* = 148) of them used more than one product (tampon plus towel) at the same time [26]. In our study, the majority of the women in the case group described their menstruation as mild or moderate before menorrhagia while severe or very severe after menorrhagia. Hence, they reported to use more than one product at the same time, their clothes, bed linens and furniture got dirty, and they experienced more pain parallel to the increase in bleeding. 

## 6. Conclusion

Menorrhagia has negative effects on women's quality of life. Therefore, quality of life of the women consulting the clinics with menorrhagia complaint should be investigated and effective approaches should be designed accordingly. 

Team of health should have thorough knowledge of menorrhagia pathophysiology and women with menorrhagia should be evaluated individually. Integrated holistic care should be provided by health professionals taking into account the physical, emotional, and social experiences. The care of the woman with menorrhagia starts with assessment phase and continues with management of the treatment and follow-up care. A detailed obstetric and gynecologic history should be obtained. Anamnesis should include the comparison of normal menstrual cycle and the current one in terms of the amount, severity, and duration of bleeding and its effects on women's life so that appropriate health enterprises could be planned. 

Future research should focus on qualitative research to understand patient's experience with menorrhagia, which will be better for effectiveness of the care and treatment provided.

## Figures and Tables

**Table 1:** Menstruation characteristics of the participants.

	Case		Control		*Z*_WMU_*	*P*
	*n*	%	*n*	%		
Severity of menstrual bleeding						
Mild	0	0	41	26.3	−10.98	0.000
Moderate	37	26.8	101	64.7		
Severe	52	37.7	11	7.1		
Very severe	49	35.5	3	1.9		
Using more than one sanitary product at the same time						
No	75	54.3	152	96.7	−9.43	0.000
Tampon and pad	6	4.4	3	1.9		
Two pads	38	27.5	2	1.4		
Diaper	19	13.8	0	0		
Getting dirty on the underwears						
Yes	138	100	129	82.7	−5.10	0.000
No	0	0	27	17.3		
Getting dirty on the clothes						
Yes	138	100	53	34.0	−11.82	0.000
No	0	0	103	66.0		
Getting dirty on the bed linens						
Yes	110	79.7	16	10.3	−12.02	0.000
No	28	20.3	140	89.7		
Getting dirty on the furniture						
Yes	68	49.3	5	3.2	−9.13	0.000
No	70	50.7	151	96.8		
Menstruation with pain						
None	18	13.0	47	29.9	−6.71	0.000
Mild	33	23.9	68	43.3		
Moderate	35	25.4	32	20.4		
Severe	27	19.6	5	3.2		
Very severe	25	18.1	5	3.2		

*Mann-Whitney *U* test.

**Table 2:** Findings about SF 36 Quality of Life Scale.

Dimensions of Quality of Life Scale	Case (*n* = 138) $\bar{X}\pm \text{SD}$	Control (*n* = 157) $\bar{X}\pm \text{SD}$	*Z*_MWU_*	*P*
Physical function	24.39 ± 5.06	28.54 ± 2.37	−9.61	0.000
Social functioning	7.26 ± 1.87	8.68 ± 1.58	−6.96	0.000
Mental health	17.21 ± 4.15	21.30 ± 3.74	−8.32	0.000
General health	15.35 ± 3.82	18.58 ± 3.18	−7.13	0.000
Role physical	1.72 ± 1.44	2.92 ± 1.47	−6.90	0.000
Role emotional	1.49 ± 1.16	2.06 ± 1.18	−4.33	0.000
Energy/Fatique	12.99 ± 3.82	16.82 ± 3.79	−7.94	0.000
Pain	6.25 ± 2.06	8.27 ± 1.80	−7.93	0.000

*Mann-Whitney *U* test.

## Appendix

### Sample Items from the Questionnaire

1. *Do you have menorrhagia (excessive uterine bleeding occurring at regular intervals)? *

1. Yes
2. No

2. *How long have you had menorrhagia? *

1. No
2. 3 months and ↓
3. 4–7 months
4. 8–11 months
5. 1-2 years
6. 2-3 years
7. 3 years ↑

3. *How many days do you have between each menstrual cycle? *

 Before Menorrhagia: (  )

 After Menorrhagia: (  )

 Control group: (  )

4. *How many times do you change pads/tampons every 24 hours? *

 Before Menorrhagia: (  )

 After Menorrhagia: (  )

 Control group: (  )

5. *Do you use more than one sanitary product at the same time? *

1. No
2. Tampon + pad
3. Two pads
4. Tampon + Two pads
5. Other …

 Before Menorrhagia: (  )

 After Menorrhagia: (  )

 Control group: (  )

6. *How many days does your menstruation last? *

1. 3 days ↓
2. 3–7 days
3. 8–10 days
4. 10 days ↑

 Before Menorrhagia: (  )

 After Menorrhagia: (  )

 Control group: (  )

7. *How do you explain severity of menstrual bleeding? *

1. Mild
2. Moderate
3. Severe
4. Very severe

 Before Menorrhagia: (  )

 After Menorrhagia: (  )

 Control group: (  )

8. *Do you experience following situations during menstruation*?

1. Yes
2. No

*Getting dirty on the underwears*

Before Menorrhagia: (  )
After Menorrhagia: (  )
Control group: (  )

*Getting dirty on the clothes*

Before Menorrhagia: (  )
After Menorrhagia: (  )
Control group: (  )

*Getting dirty on the bed linens*

Before Menorrhagia: (  )
After Menorrhagia: (  )
Control group: (  )

*Getting dirty on the furniture*

Before Menorrhagia: (  )
After Menorrhagia: (  )
Control group: (  )

9. *Do you have dysmenorrhea? *

1. None
2. Mild
3. Moderate
4. Severe
5. Very severe

 Before Menorrhagia: (  )

 After Menorrhagia: (  )

 Control group: (  )

10. *Do you know if any woman (mother, sister, or other) in your family has or has had menorrhagia? *

1. Yes
2. No

11. *Has anyone of your relatives (women and men) problems with any other kind of bleeding? *

1. Yes
2. No
